# Cytotoxic and Apoptotic Effects of Pinostilbene and Bortezomib Combination Treatment on Human Multiple Myeloma Cells

**DOI:** 10.3390/ijms241612590

**Published:** 2023-08-09

**Authors:** Anna Staskiewicz, Erica Wong, Michael Tucker, Riya Farhin, Jonathan Park, Rana Saade, Tina Alkhazali, Tu Dang, Xinyu Wang

**Affiliations:** Department of Pharmaceutical Sciences, College of Pharmacy, Philadelphia College of Osteopathic Medicine–Georgia Campus, Suwanee, GA 30024, USA; as0735@pcom.edu (A.S.);

**Keywords:** multiple myeloma, bortezomib, resveratrol, pinostilbene, piceatannol, synergism, cytotoxicity, apoptosis, bone marrow microenvironment, oxidative stress

## Abstract

Multiple myeloma (MM) is a cancer of plasma cells in the bone marrow characterized by bone lesions, hypercalcemia, anemia, and renal failure. Bortezomib (BTZ), a common treatment for MM, is a proteasome inhibitor that induces apoptosis in MM cells. However, high doses of BTZ can be very toxic, signifying a need for a synergistic drug combination to improve treatment efficacy. Resveratrol (RES), a phenolic compound found in grapes, has been shown to inhibit MM cell growth. We sought to identify a synergistic combination of BTZ with a RES derivative and analyze the effects on reducing viability and inducing apoptosis in human MM cells. BTZ as well as RES and its derivatives pinostilbene (PIN) and piceatannol (PIC) decreased MM cell viability in a dose- and time-dependent manner and increased expression of cleaved proapoptotic proteins poly(ADP-ribose) polymerase 1 (PARP1) and caspase-3 in a dose-dependent manner. The combination of 5 nM BTZ and 5 μM PIN was identified to have synergistic cytotoxic effects in MM RPMI 8226 cells. MM RPMI 8226 cells treated with this combination for 24 h showed increased cleaved PARP1 and caspase-3 expression and higher percentages of apoptotic cells versus cells treated with the individual compounds alone. The treatment also showed increased apoptosis induction in MM RPMI 8226 cells co-cultured with human bone marrow stromal HS-5 cells in a Transwell model used to mimic the bone marrow microenvironment. Expression of oxidative stress defense proteins (catalase, thioredoxin, and superoxide dismutase) in RPMI 8226 cells were reduced after 24 h treatment, and cytotoxic effects of the treatment were ameliorated by antioxidant N-acetylcysteine (NAC), suggesting the treatment impacts antioxidant levels in RPMI 8226 cells. Our results suggest that this combination of BTZ and PIN decreases MM cell viability synergistically by inducing apoptosis and oxidative stress in MM cells.

## 1. Introduction

Multiple myeloma (MM) is a malignancy affecting plasma cells within the bone marrow. MM remains without a cure, and 34,470 people in the US were predicted to be diagnosed with this cancer in 2022 [1]. MM typically develops from a monoclonal gammopathy of undetermined significance (MGUS), characterized by the presence of M-proteins in the blood, which are abnormal monoclonal proteins secreted by these malignant plasma cells [2]. MM is distinguished by four main symptoms: anemia, bone lesions, hypercalcemia, and renal failure [3]. MM cell growth causes suppression of erythropoiesis, resulting in decreased levels of red blood cells [4]. MM cells also directly interact with osteocytes, which consequently upregulate bone resorption by osteoclasts and downregulate bone formation by osteoblasts [5]. This imbalance in bone remodeling contributes to hypercalcemia due to increased levels of calcium being released from bone into the blood [6]. Hypercalcemia is a major contributor to renal failure as the altered blood osmolality affects the ability of the kidneys to concentrate urine; renal function is also impaired because of the accumulation of M-proteins obstructing the renal tubular system [7].

Treatments for MM include proteasome inhibitors, immunomodulators, monoclonal antibodies, corticosteroids, histone deacetylase inhibitors, and radiation therapy [8,9]. Autologous stem cell transplantation (SCT) is recommended for eligible, newly diagnosed individuals without comorbidities and involves induction therapy, high-dose chemotherapy, SCT, and maintenance using the immunomodulator and angiogenesis inhibitor lenalidomide [10]. Although novel treatments have increased the survival of people with MM, virtually all patients will have a relapse, making treatment more complex [11]. In MM, the bone marrow microenvironment, which includes plasma cells, bone marrow stromal cells (BMSCs), osteoclasts, and osteoblasts, becomes altered in a manner that supports MM cell survival, contributing to increased resistance to chemotherapeutic drugs such as bortezomib (BTZ) [12]. 

BTZ is a boron containing molecule that was the first approved proteasome inhibitor used for treating MM and was one of the treatments that marked the transition to better patient outcomes [13]. BTZ targets the 26S proteasome through inhibiting the threonine residue, preventing protein degradation by the proteasome. The resulting accumulated protein within the cells results in endoplasmic reticulum (ER) stress [14]. BTZ induces apoptosis of MM cells through ER stress and has been shown to inhibit the activation of nuclear factor-kappa B (NF-ĸB), a transcription factor that increases MM cell adhesion to stromal cells and induces a number of growth and angiogenesis factors [15]. The apoptotic pathway is normally inhibited in cancer cells, resulting in prolonged survival and overgrowth of these cells; thus, many anti-cancer treatments target and activate this pathway. Despite their inhibition of apoptosis, cancer cells are more sensitive to apoptosis-inducing treatments than normal cells due to oncogenic and environmental stressors [16]. Although BTZ is often used to target MM cells, the treatment has been demonstrated to have adverse effects including gastrointestinal issues, fatigue, thrombocytopenia, and peripheral neuropathy [17]. Dosage from BTZ treatment must be limited to manage the severity of its adverse effects, with peripheral neuropathy particularly requiring careful monitoring to identify when to lower the maximum recommended 1.3 mg/m^2^ dosage. [18]. One option to address this issue is to identify synergistic combinations of BTZ with other compounds, which show anti-myeloma effects but less toxicity in normal cells than BTZ. Identifying a synergistic combination would assist in maintaining a safe dose while enhancing desired effects on targeted cancer cells.

Resveratrol (RES), a phenolic compound found in grapes, has a variety of applications due to its antioxidative, anti-inflammatory, anti-cancer, and immunomodulatory properties [19]. Piceatannol (PIC), a hydroxylated RES derivative, is a known Syk tyrosine kinase inhibitor with anti-cancer and anti-inflammatory effects [20]. Both RES and PIC have been shown to inhibit MM cell growth and induce apoptosis in MM cells [21,22]. RES’s mono-methoxylated derivative pinostilbene (PIN) inhibits the growth of various cancer cells, including colon and oral cancer cells [23,24]. However, PIN’s effect on MM cells has not been studied yet. Previous studies have shown that RES in combination with other anti-MM compounds such as BTZ, carfilzomib (CFZ), and rapamycin can have a synergistic effect, further increasing the proportion of MM cells undergoing apoptosis compared to a treatment of those compounds alone [25,26,27]. RES causes apoptosis in human colon and ovarian cancer cells through inducing autophagy by stimulating reactive oxygen species (ROS) production [28,29]. Regarding MM specifically, RES induces apoptosis in BTZ-resistant MM cells partially by inhibiting the Hedgehog signaling pathway [30]. Furthermore, the addition of RES to the treatment of CFZ, a second-generation proteasome inhibitor, further induces MM cell apoptosis by oxidative stress [26]. 

Taking into consideration the efficacy of the already implemented MM drug BTZ, the potential that RES derivatives show in targeting MM cells and their possibly different mechanisms of action, there is potential for the two drug types to be synergistic for cytotoxic effects on MM cells. In this study, we aimed to compare the cytotoxic effects of RES and derivatives PIN and PIC in MM cells and identify a combination treatment of a RES derivative with BTZ that synergistically reduces MM cell viability. We sought to assess the combination’s effects on apoptosis induction in MM cells both cultured alone and co-cultured with BMSCs, using a Transwell model to mimic the bone marrow microenvironment. We additionally aimed to assess the treatment’s effects on oxidative stress induction in MM cells.

## 2. Results

### 2.1. Resveratrol, Pinostilbene, Piceatannol, and Bortezomib Inhibit Multiple Myeloma RPMI 8226 Cell Growth 

**Figure 1:**
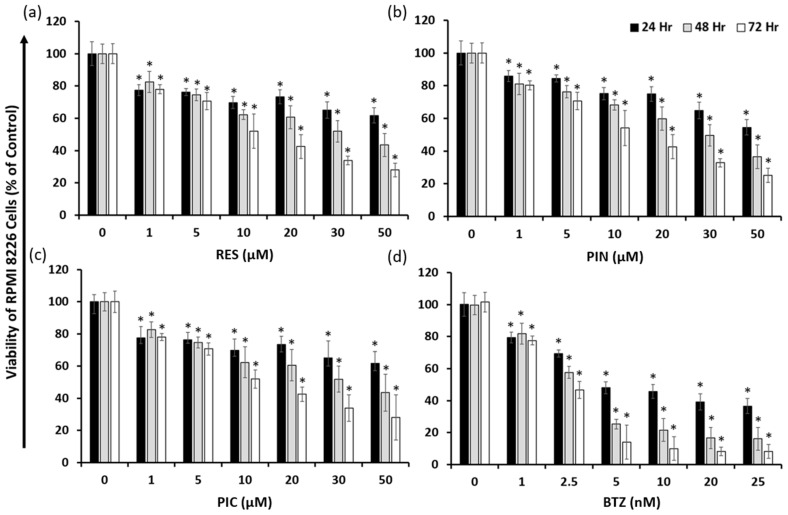
Cell viability of MM RPMI 8226 cells treated with RES (**a**), PIN (**b**), PIC (**c**), and BTZ (**d**). Fluorescence values were normalized to the 0 μM treatment (0.05% DMSO vehicle control for RES, PIN, and PIC; 0.00005% DMSO for BTZ) which signifies 100% cell viability. All compounds show demonstrated dose- and time-dependent effects on reducing RPMI 8226 cell viability within 72 h. *: *p* < 0.05 indicates significant difference vs. vehicle control.

With the aim to study potential drug treatments for MM, we first assessed the cytotoxic effects of RES, its derivatives (PIN and PIC), and BTZ in MM RPMI 8226 cells. RES and its derivatives PIN and PIC demonstrated overall dose- and time-dependent effects in reducing RPMI 8226 viability (Figure 1a–c). As concentrations of each compound increased from 0 μM to 50 μM, the viability of treated RPMI 8226 cells decreased, with all the concentrations being significantly different compared to the vehicle control. For the results at the 24, 48, and 72-h time points, the viability of the cells tended to decrease as the cells were exposed to the treatment of each compound for a longer duration, with the exception of the low concentrations of 1 μM RES and 1 μM PIC. To compare the potencies of the RES derivatives, IC_50_ values were calculated from the individual compound viability data (Table 1). RES had the highest IC_50_ value at 24 and 48 h and a value similar to those of PIN and PIC at 72 h. PIC had the lowest IC_50_ value at 24 and 48 h. PIN showed IC_50_ values similar to RES at each time point. BTZ showed an overall dose- and time-dependent effect in reducing RPMI 8226 viability from tested 2.5 nM to 25 nM concentrations (Figure 1d). From the 1 nM to 25 nM BTZ treatments, the viability of RPMI 8226 cells decreased as the treatment concentration increased. At each tested concentration, with the exception of 1 nM BTZ, the viability of the cells decreased over time. BTZ had IC_50_ values over 1000 times lower than the RES derivatives at all time points (Table 1). At 72 h, the IC_50_ values for each of the RES derivatives were approximately 13 μM while the value for BTZ was around 2 nM (Table 1). Overall, the RES derivatives and BTZ showed anti-myeloma effects in the RPMI 8226 cell line, with BTZ being much more cytotoxic and potent in comparison to the RES derivatives. 

### 2.2. Resveratrol Derivatives Have Varying Cytotoxic Effects on Different Human MM Cell Lines

**Figure 2:**
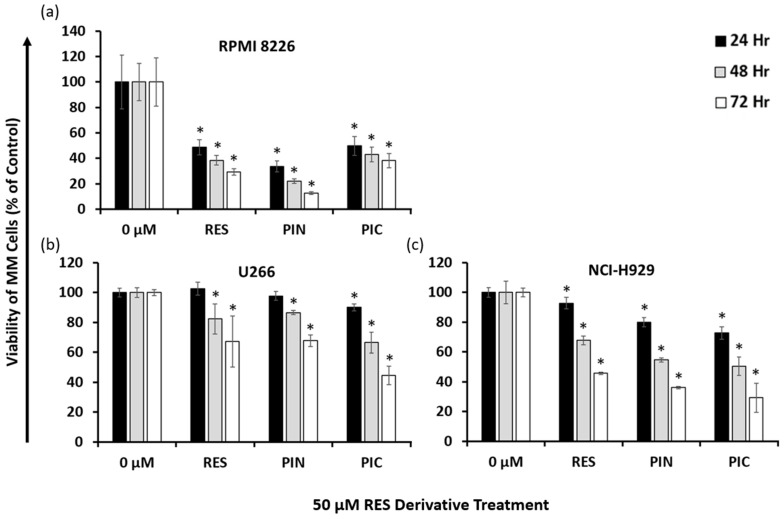
Cell viability effects of 50 μM RES and derivatives in MM cell lines RPMI 8226 (**a**), U266 (**b**), and NCI-H929 (**c**). Fluorescence values were normalized to the 0 μM treatment (0.05% DMSO vehicle control) which signifies 100% cell viability. RES derivatives exhibited time-dependent cytotoxicity in all tested MM cell lines and were most cytotoxic to the RPMI 8226 cell line. *: *p* < 0.05 indicates significant difference vs. vehicle control.

To confirm the common cytotoxic efficacies of RES and its derivatives PIN and PIC on MM cells, cells from MM cell lines RPMI 8226, U266, and NCI-H929 were treated with 50 μM of each compound, and viability was assessed 24, 48, and 72 h following treatment. In the RPMI 8226 cell line, all three of the tested compounds significantly reduced the viability of these cells (Figure 2a). PIN showed the greatest cytotoxic effect across tested time points, while PIC had the least effect. Conversely, PIC was the compound with the greatest cytotoxic effect on U266 cells, while RES and PIN had similar effects to one another (Figure 2b). In NCI-H929 cells, PIN and PIC exhibited similar inhibitory effects on cell viability, and RES was the least cytotoxic (Figure 2c). Across three MM cell lines, RES derivatives showed the greatest cytotoxic effects on RPMI 8226 cells, followed by their effects on NCI-H929 cells, and lastly on U266 cells, which showed the least cytotoxicity. PIN was the most cytotoxic RES derivative in RPMI 8226 cells, while PIC was the most cytotoxic in U266 and NCI-H929 cells. Apart from RES and PIN treatments in the U266 cell line that showed significant cytotoxicity starting at 48 h following treatment, all the tested compounds had significant effects on cell viability starting at 24 h. Taken together, our data show RES, PIN, and PIC are cytotoxic to MM cells at 50 μM concentrations and vary in their efficacies across cell lines.

### 2.3. A Combination of 5 μM PIN and 5 nM BTZ Is Synergistically Cytotoxic to MM Cells and Is Minimally Toxic to Normal Human Peripheral Blood Mononuclear Cells (PBMC)

**Figure 3:**
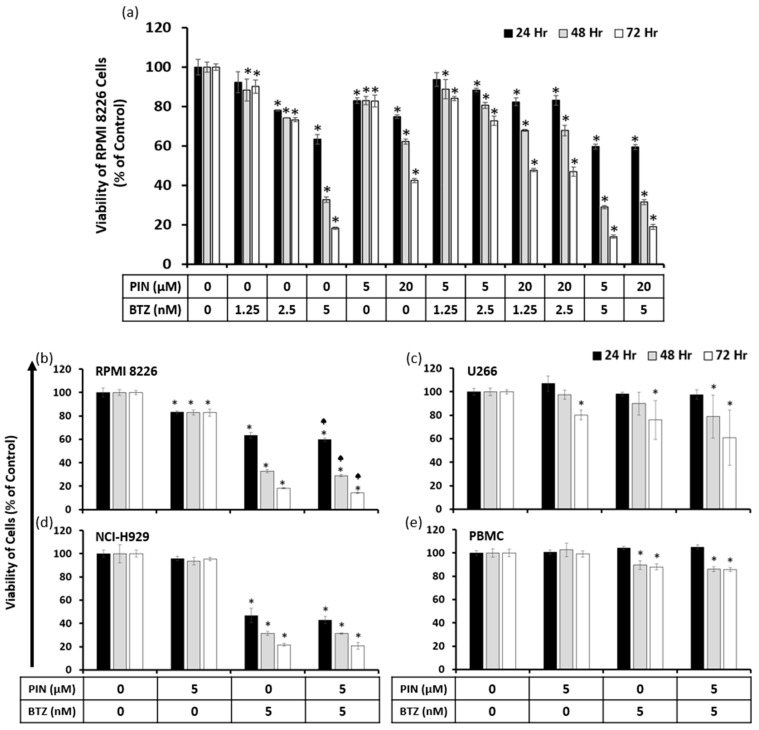
Cytotoxic effects of PIN and BTZ on MM and normal PBMC cells. Viability of RPMI 8226 cells was measured after individual and combination treatment with PIN and BTZ at various concentrations (**a**). Fluorescence values were normalized to the 0 μM PIN/0 nM BTZ treatment (0.05% DMSO vehicle control) which signifies 100% cell viability. Combinations of PIN and BTZ exhibited varying cytotoxic effects in RPMI 8226 cells, with the combination of 5 μM PIN/5 nM BTZ demonstrating the greatest cytotoxicity out of the tested combinations. Cytotoxic effects of the 5 μM PIN/5 nM BTZ treatment were assessed in MM cell lines RPMI 8226 (**b**), U266 (**c**), and NCI-H929 (**d**) as well as in normal PBMC (**e**) cells. The combination treatment of 5 μM PIN/5 nM BTZ was significantly more cytotoxic to RPMI 8226 cells than 5 nM BTZ alone. The combination was slightly more cytotoxic than BTZ in U266 and NCI-H929 cells but not statistically significant. The treatment showed minimal cytotoxic effects in normal PBMC cells. *: *p* < 0.0001 indicates significant difference vs. vehicle control. ♠: *p* < 0.05 indicates significant difference of the combination PIN/BTZ group vs. the 5 nM BTZ group.

Based on preliminary screenings for RPMI 8226 cytotoxicity by RES derivatives with BTZ, PIN demonstrated the greatest potential for synergism when combined with BTZ. Considering the screenings and previously calculated IC_50_ values of the compounds in RPMI 8226 cells, we selected the 5 and 20 μM concentrations of PIN with 1.25, 2.5 and 5 nM concentrations of BTZ to confirm any synergistic effects. Effects of PIN and BTZ together were assessed with viability assays in RPMI 8226 cells, where the cells were treated with the individual compounds in addition to the combination treatments. The 5 μM PIN/5 nM BTZ combination showed the greatest cytotoxic effect out of the tested combinations (Figure 3a). All tested combination treatments were significantly different from the vehicle control except for 5 μM PIN/1.25 nM BTZ at the 24 h time point. To determine synergism, the CI values were calculated from this viability data using CompuSyn (Ver 1.0) software (ComboSyn, Inc., Paramus, NJ, USA), and the 5 μM PIN/5 nM BTZ combination had CI values of less than 1 at all tested time points (Table 2). This indicates a synergistic effect of the combination in reducing RPMI 8226 cell viability at all tested time points. The other combinations had calculated CIs of greater than 1, indicating antagonistic effects. We selected the 5 μM PIN and 5 nM BTZ combination for further assessment as a synergistic treatment, which is referenced as the PIN/BTZ combination through this study.

To evaluate the effectiveness of the PIN/BTZ combination in MM cells, cells from three MM cell lines were treated with this combination, and viability was assessed after 24, 48, and 72 h. At all tested time points, the RPMI 8226 cell viability from the PIN/BTZ treatment was significantly different from the viability in cells treated with 5 nM BTZ alone (Figure 3b). In the case of U266 and NCI-H929 cells, the effects of the combination treatment at all time points showed a trend of slightly increased cytotoxic effects than those of the BTZ treatment alone, but these differences were not statistically significant (Figure 3c,d). Across the MM cell lines tested, 5 nM BTZ was more cytotoxic in comparison to 5 μM PIN, and the PIN/BTZ combination tended to reduce the viability the most out of these treatment groups.

To observe the effects of PIN/BTZ on normal PBMC cells, these cells were treated with the combination treatment as well as its individual components. The 5 μM PIN treatment did not have a significant effect on PBMC viability, while 5 nM BTZ and the PIN/BTZ combination decreased the viability to approximately 85% after 48 and 72 h (Figure 3e). In comparison to the MM cell lines, the combination’s cytotoxic effect is much lower in PBMC cells, suggesting the treatment selectively targets MM cells.

### 2.4. The 5 μM PIN and 5 nM BTZ Combination Treatment Increases Percentages of Apoptotic RPMI 8226 Cells

**Figure 4:**
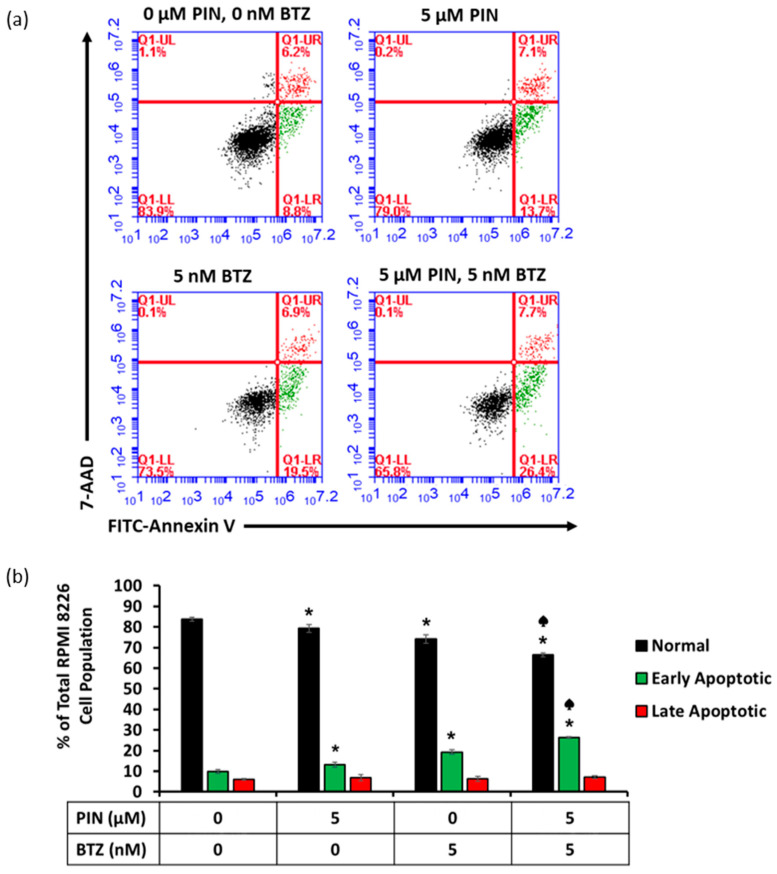
Apoptosis induction in MM RPMI 8226 cells 24 h after 5 μM PIN, 5 nM BTZ, and PIN/BTZ combination treatment. Gating for flow cytometric analysis was used to distinguish viable cells (Annexin V-, 7-AAD-, black), early apoptotic cells (Annexin V+, 7-AAD-, green), and late apoptotic cells (Annexin V+, 7-AAD+, red) following treatment (**a**). Percentages of apoptotic cells from three replicates are shown in (**b**). The increased percentage of early apoptotic cells supports apoptosis induction as one mechanism targeted by the treatment of PIN and BTZ. The PIN/BTZ combination treatment induces more apoptosis of RPMI 8226 cells than BTZ and PIN treatment alone, further confirming the synergistic effects. *: *p* < 0.05 indicates significant difference vs. 0 μM PIN/0 nM BTZ (0.05% DMSO vehicle control). ♠: *p* < 0.05 indicates significant difference of the 5 μM PIN/5 nM BTZ group vs. the 5 nM BTZ group.

RPMI 8226 cells were treated for 24 h with PIN/BTZ combination, stained with 7-AAD and FITC-Annexin V, and analyzed using flow cytometry to determine apoptosis induction by this treatment. The data from these treated RPMI 8226 cells showed increased percentages of early apoptotic cells and decreased percentages of viable cells 24 h following PIN/BTZ combination treatment in comparison to 5 μM PIN or 5 nM BTZ treatment (Figure 4a,b). In the cell population treated with the combination treatment, the percentage of viable cells was significantly lower than that in the 5 nM BTZ-treated cells, and the percentage of cells in early apoptosis was significantly greater than that in the 5 nM BTZ-treated cells. There was no significant difference in the percentage of cells in late apoptosis across the treatment groups. These data suggest the PIN/BTZ combination targets MM cells through inducing apoptosis.

### 2.5. The 5 μM PIN and 5 nM BTZ Combination Treatment Increases Proapoptotic Protein Expression in MM RPMI 8226 Cells Cultured Individually and Co-Cultured with Human Bone Marrow Stromal HS-5 Cells

**Figure 5:**
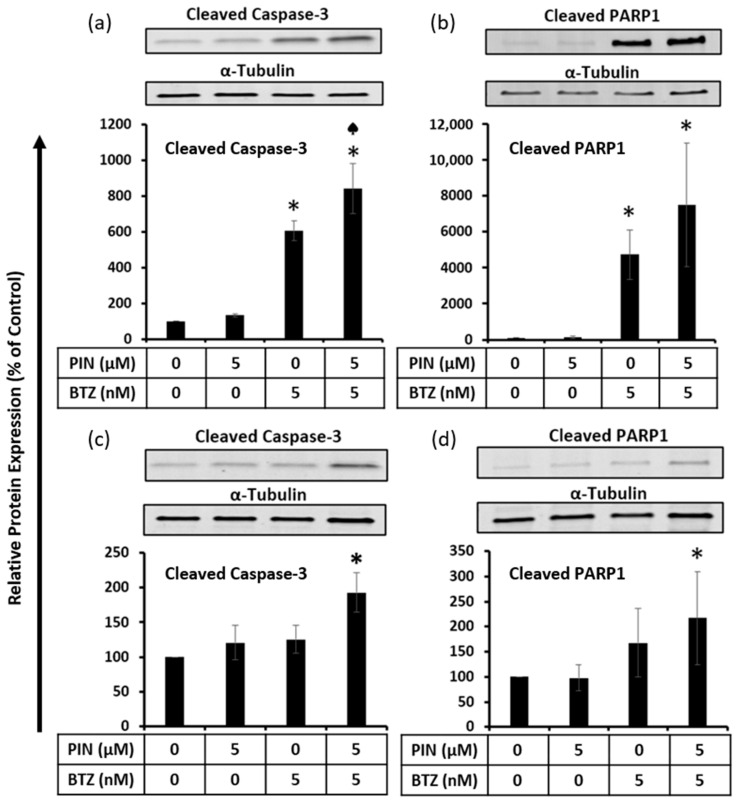
Apoptosis induction confirmed through Western blotting by evaluating expression of cleaved caspase-3 and cleaved PARP1 expression in individually cultured RPMI 8226 cells (**a**,**b**) and RPMI 8226 cells co-cultured with HS-5 cells in a Transwell model (**c**,**d**). Band intensity quantification was normalized to the α-tubulin loading control. Treatment group values were normalized to the 0 μM PIN/0 nM BTZ group treatment (0.05% DMSO vehicle control), which signifies 100% protein expression. The 5 μM PIN/5 nM BTZ treatment showed an overall increasing trend in the expression of both proapoptotic proteins in RPMI 8226 cells compared to its individual components alone. This trend was seen in both RPMI 8226 cells cultured alone and in those co-cultured with HS-5 cells, although to a lesser magnitude in the co-cultured cell model. *: *p* < 0.05 indicates significant difference vs. 0 μM PIN/0 nM BTZ (0.05% DMSO vehicle control). ♠: *p* < 0.05 indicates significant difference of the PIN/BTZ group vs. the 5 nM BTZ group.

To confirm the apoptosis induction observed from the flow cytometry data, proapoptotic protein expression in PIN/BTZ-treated RPMI 8226 cells was assessed using Western blotting. In relation to cleaved caspase-3 expression, the protein expression from the combination treatment was significantly greater than the expression from 5 nM BTZ alone, which is the more potent component of the treatment versus the 5 μM PIN component (Figure 5a). The trend seen for cleaved PARP1 expression suggests that the PIN/BTZ treatment increases cleaved PARP1 expression in RPMI 8226 cells more than the 5 μM PIN and 5 nM BTZ components alone (Figure 5b). In RPMI 8226 cells co-cultured with HS-5 cells using the Transwell model, a similar trend was seen where the combination treatment seemed to increase the cleaved caspase-3 and cleaved PARP1 expression more than 5 μM PIN or 5 nM BTZ alone (Figure 5c,d). However, the effects on proapoptotic protein expression in RPMI 8226 cells co-cultured with bone marrow stromal HS-5 cells were not as drastic as those seen in the RPMI 8226 cells cultured alone. These data show that even with the increased drug resistance experienced by MM cells co-cultured with BMSCs, the treatment induces apoptosis in the MM cells.

### 2.6. The 5 μM PIN and 5 nM BTZ Combination Treatment Affects the Oxidative State of MM RPMI 8226 Cells

**Figure 6:**
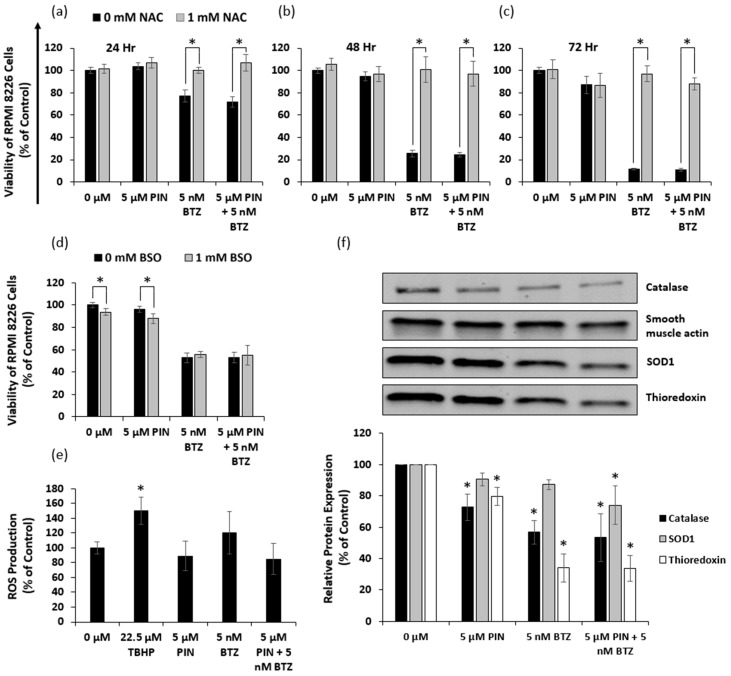
Analysis of PIN/BTZ induction of oxidative stress in RPMI 8226 cells. Cell viability of RPMI 8226 cells pretreated with 0 mM NAC or 1 mM NAC and treated with PIN/BTZ for 24 (**a**), 48 (**b**), and 72 h (**c**). Cell viability of RPMI 8226 cells pretreated with 0 mM BSO or 1 mM BSO and treated with PIN/BTZ for 24 h (**d**). Assessment of ROS production following DCFDA staining of RPMI 8226 cells and 24 h treatment with PIN/BTZ (**e**). Western blotting for catalase, SOD1, and thioredoxin expression in RPMI 8226 cells treated with PIN/BTZ for 24 h (**f**). NAC treatment rescued cells from 5 nM BTZ and 5 μM PIN/5 nM BTZ treatment at tested time points. BSO reduced RPMI 8226 cell viability only in the DMSO control and 5 μM PIN treatment groups. PIN, BTZ, and the combination treatment showed no significant difference in ROS production versus the DMSO control, while the positive control TBHP showed significantly increased ROS levels. PIN and BTZ decreased expression of catalase, SOD1, and thioredoxin in the cells, with the 5 μM PIN/5 nM BTZ showing the greatest decrease in protein expression. *: *p* < 0.05 indicates significant difference vs. 0 mM NAC- or BSO-treated group (**a**–**d**) or vs. 0 μM (0.05% DMSO vehicle control) (**e**,**f**).

N-acetylcysteine (NAC) itself is a precursor of the antioxidant glutathione, which scavenges ROS, while buthionine sulfoximine (BSO) is known to inhibit glutathione biosynthesis. We treated RPMI 8226 cells with 1 mM NAC or 1 mM BSO prior to treating the cells with PIN and BTZ. To assess the possibility of oxidative stress induced by PIN/BTZ, we sought to observe whether NAC treatment rescues the cells and whether BSO increases cytotoxic effects. Across all tested time points, we observed there to be a significant difference in viability between NAC-treated cells and NAC-untreated cells for those treated with BTZ or the PIN/BTZ combination treatment (Figure 6a–c). We saw no significant difference between NAC treated and untreated cells for the PIN treatment group (Figure 6a–c). Interestingly, BSO did not increase cytotoxic effects in the BTZ and PIN/BTZ groups, but did slightly decrease viability in the control and PIN groups (Figure 6d).

RPMI 8226 cells were also stained with DCFDA to estimate relative levels of ROS production from treatment with PIN and BTZ. The positive control of 22.5 μM tert-Butyl hydroperoxide (TBHP) showed a significant increase in ROS production (Figure 6e). There were no significant differences between the DMSO control group versus the PIN, BTZ, and PIN/BTZ treatments, though there was a trend of slightly increased ROS production by BTZ and decreased ROS production by PIN and PIN/BTZ (Figure 6e). The PIN may counteract the ROS production by BTZ in the combination treatment.

We also assessed effects of PIN and BTZ on protein expression of catalase, superoxide dismutase (SOD1), and thioredoxin in RPMI 8226 cells. These proteins act as ROS scavengers. Both catalase and thioredoxin expression were decreased across treatment groups versus the DMSO control, while SOD1 expression also showed a decrease though only significantly in the PIN/BTZ-treated group (Figure 6f). Of the treatment groups, PIN showed the greatest expression of these proteins while the PIN/BTZ combination showed the least expression. RPMI 8226 cells treated with the PIN/BTZ combination were saved by NAC treatment and also significantly reduced the expression of oxidative stress defense proteins, suggesting the treatment targets cells via induction of oxidative stress through lowering antioxidant levels.

## 3. Discussion

In the U.S., MM is the second most common malignancy of the blood [31]. MM’s challenging symptoms significantly impact people’s lives and survival, but MM is a disease that remains without a cure. Survival rates have been increasing with the introduction of novel treatments [32], but significant progress still needs to be made. As almost every person who recovers from MM relapses, studying potential treatment options is important to hopefully improve outcomes for patients. Currently, BTZ is used clinically to treat MM as the first-generation proteasome inhibitor. It inhibits the protein-degrading function of the proteasome, resulting in protein accumulation that leads to ER stress induced apoptosis in MM cells [14,15]. For this project, we focused on investigating whether BTZ treatment could be improved upon with the addition of a RES derivative. RES itself has been shown to have cytotoxic effects in a variety of cancer cells in addition to MM cells, including breast, prostate, and pancreatic cancer cells [33,34,35]. Several studies have pinpointed oxidative stress as a mechanism RES targets in cancer cells [34,35]. RES downregulates NF-κB and STAT3 in MM cells, which are key targets in cancer therapy, and potentiates the apoptosis induced by BTZ in MM cells [27]. However, RES has been shown to have very low bioavailability [36], and several of its derivatives have been studied in the interest of identifying more effective novel treatments. A combination of RES and BTZ treatment has been shown to have synergistic anti-myeloma effects in vitro, but when applied in vivo led to some adverse renal effects [30,37]. The cytotoxic effects of RES derivatives have been studied in MM cells and cancer cells in general [22,24,29]. Due to differences in how RES derivatives and BTZ can target MM cells, we hypothesized that a combination of a RES derivative and BTZ can be synergistically cytotoxic to MM cells.

Our data suggest that RES, PIN, and PIC all have dose- and time-dependent effects on reducing the viability of RPMI 8226 cells (Figure 1). Other studies have demonstrated cytotoxic effects of RES and PIC in MM cells [22,27], and we show PIN is an additional RES derivative exhibiting MM cytotoxicity. This highlights PIN as a compound which would be beneficial to further research in MM as well as other cancer cells. BTZ showed dose-and time-dependent cytotoxicity in RPMI 8226 cells, with a calculated thousand-fold lower IC_50_ compared to the RES derivatives (Table 1). Our results support previously observed dose- and time-dependent RPMI 8226 cytotoxicity of BTZ and demonstrate BTZ IC_50_ values within a similar range [38,39]. Of the three MM cell lines tested (RPMI 8226, U266, and NCI-H929), RES derivatives overall exhibited the greatest cytotoxicity in RPMI 8226 cells. Additionally, the relative efficacies of 50 μM RES derivatives varied across MM cell lines (Figure 2). PIN was the most cytotoxic of the derivatives in RPMI 8226 cells, while PIC was the most cytotoxic compound in U266 cells. As for NCI-H929 cells, the cytotoxic effects of PIN and PIC were similar and surpassed those of RES. Current literature shows varying cytotoxic effects of RES derivatives in different MM cell lines. Similar to our results, a study by Schmeel et al. showed PIC to have greater cytotoxic effects in RPMI 8226 versus U266 cells [22], while Sun et al. observed similar RES efficacy in RPMI 8226 and U266 cells [40]. The original cells of the three MM cell lines were initially collected from donors of different backgrounds and stages of MM, with the cells differing in characteristics such as cell surface markers [41]. The genetic variety of the MM cell lines leads to the variation in treatment efficacy for in vitro experiments, which then translates to different treatment outcomes for different people with the disorder. 

Following the study of the compounds’ effects individually, we sought to identify a synergistic combination of one RES derivative with BTZ and assess its effects on MM cell viability and apoptosis. To search for potential synergistic drug combinations, treatments of RES derivatives in combination with BTZ were screened for their cytotoxic effects in RPMI 8226 cells. Our study led to a combination of PIN and BTZ, and synergistic effects were confirmed using CI values. A combination treatment of 5 μM PIN and 5 nM BTZ treatment showed the greatest cytotoxic effect on RPMI 8226 cells of the tested combinations (Figure 3a) and was also confirmed by CompuSyn (Ver 1.0) as synergistic at all tested time points (Table 2). Three MM cell lines were treated to confirm the PIN/BTZ treatment’s anti-myeloma effects. For RPMI 8226 cells, the combination treatment was significantly more cytotoxic than the more effective component of 5 nM BTZ at all time points tested. A similar trend was seen for the U266 and NCI-H929 cell lines (Figure 3). Our data support this particular combination of PIN/BTZ as being synergistic in reducing the viability of MM RPMI 8226 cells, although the extent of synergism seems to differ in the other two cell lines. Interestingly, BTZ has been shown to have similar effects in RPMI 8226 and U266 cells [42], while our study showed differing cytotoxic effects from PIN/BTZ in these two cell lines. Rather, we observed NCI-H929 cells to have similar cytotoxic effects to RPMI 8226 cells from PIN/BTZ treatment. This may suggest the interactions between PIN and BTZ differentially affect MM cell viability across cell lines. 

The purpose for studying synergistic treatment is to minimize the concentration of BTZ needed to effectively target MM and consequently reduce non-MM toxic effects such as peripheral neuropathy, one of the main side effects of BTZ [17]. To confirm PIN/BTZ treatment selectively targets MM cells, PBMC cell viability was also tested to determine whether the combination impacts normal human blood cells, which would present alongside MM cells in vivo. These cells include blood cells with round nuclei, consisting of lymphocytes, monocytes, and dendritic cells, with lymphocytes making up 70–90% of the cells [43]. As MM are malignant b-lymphocytes, we studied effects on PBMC cells as they include the malignant cells’ normal counterparts in addition to other cell populations found in the blood. PBMC cell viability did not drop below 85% from treatment (Figure 3e). We would expect the BTZ component to exhibit some toxicity in these cells, as BTZ has been shown to increase NF-κB activity in PBMC cells [44]. The PIN component is likely to have little cytotoxic effect on PBMC cells as derivatives RES and PIC are selectively cytotoxic to malignant cells and minimally affect normal PBMC cell viability [22,45]. Our data suggest that the combination treatment selectively inhibits MM cell growth in comparison to normal cells, which is vital in the consideration of potential MM drug treatments. 

Apoptosis induction in MM cells was assessed due to increased sensitivity of cancer cells to apoptosis-targeting treatments [16]. Flow cytometric analysis showed a decrease of the viable cell population and an increase in early apoptotic cells with the individual 5 μM PIN and 5 nM BTZ treatments (Figure 4). Cells treated with the combination PIN/BTZ showed even greater apoptotic effects. We additionally evaluated the expression of proapoptotic proteins in treated RPMI 8226 cells. Caspases make up a family of cysteine aspartate proteases which play an integral role in apoptotic cell death [46]. Initiator caspases such as caspase-8 and -9 are activated via dimerization and cleave executioner caspases, which exhibit proteolytic activity towards a variety of substrates to break down cellular components [47]. Caspase-3 is an executioner caspase with a highly involved role in apoptosis and cleaves PARP1 as one of its many downstream targets [48,49]. Due to their roles in apoptosis, we blotted for the cleaved forms of caspase-3 and PARP1. We observed greater protein expression of the cleaved proapoptotic proteins in 5 nM BTZ-treated cells versus 5 μM PIN-treated cells (Figure 5a,b), which aligns with the trend seen for the cytotoxic effects of these treatments. The combination PIN/BTZ showed an even further increase in these proteins’ expression. These data suggest the combination treatment induces apoptosis in RPMI 8226 cells to a greater extent than either of the individual components.

**Figure 7:**
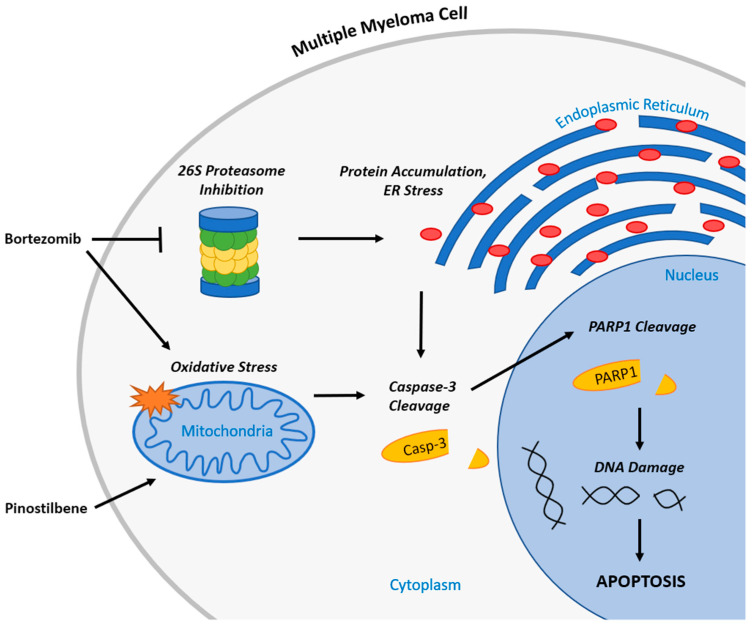
Schematic illustration of PIN and BTZ combination’s proposed mechanism of action in targeting MM cells.

As we show in a schematic illustration of the potential anti-myeloma mechanism of BTZ and PIN (Figure 7), MM apoptotic induction from PIN/BTZ treatment likely occurs from the proteasome inhibition and oxidative stress induced by BTZ [14,15,50]. We expected PIN to also induce oxidative stress as PIN is a derivative of RES, which is shown to cause oxidative stress in several cancer cells including MM cells [26,28,34,35]. The dose of 5 μM PIN used in our study showed minimal effects on ROS production in RPMI 8226 cells, and treated cells were not saved by ROS inhibitor NAC (Figure 6). However, PIN did decrease expression of each tested oxidative stress defense protein (catalase, thioredoxin, and SOD1). These proteins’ expression decreased in a similar trend to cytotoxicity and apoptosis induction data, with PIN treatment decreasing overall protein expression compared to control, followed by a further decrease in the BTZ treatment and then the PIN/BTZ treatment. Downregulation of these antioxidant enzymes by PIN/BTZ sensitizes MM cells to oxidative stress, especially considering MM cells have increased ROS levels compared to normal plasma cells [51]. ROS exposure has been shown to downregulate catalase expression in human hepatoma cells [52], and another study linked low catalase expression to MM cells sensitive to ROS generation by parthenolide treatment [53]. Inhibition of thioredoxin has been shown to cause ROS-dependent apoptosis in MM cells and sensitizes MM cells to certain treatments including NF-ĸB inhibitors [54]. Similar to our results, Nerini-Molteni et al. observed NAC to rescue RPMI8226 cells, as well as U266 cells, from BTZ treatment [55]. Though our data showed BTZ and PIN/BTZ effects to be rescued by NAC, we did not observe a difference in MM cell ROS production between these treatments and the control group. Interestingly, MM cells have been shown to undergo cell death by either increasing or decreasing ROS levels [51]. The NAC rescuing effects and downregulated oxidative stress defense proteins suggest oxidative stress as another mechanism targeted by PIN/BTZ in RPMI 8226 MM cells. Studies with various concentrations of PIN would more clearly confirm whether PIN itself induces or ameliorates oxidative stress in MM cells and whether it does so in a dose-dependent manner.

Interactions between BMSCs, osteoblasts, osteoclasts, endothelial cells, and MM cells alter the bone marrow microenvironment, largely contributing to drug resistance exhibited by MM cells in vivo [49]. BMSC secretions are major contributors to this resistance. A previous study showed exosomes from BMSCs increased proliferation and viability of MM cells as well as increased MM cell resistance to BTZ treatment [50]. Taking into consideration the significance of such interactions on assessing potential drug treatment, we assessed the apoptotic effects of PIN/BTZ on RPMI 8226 cells when co-cultured with human BMSCs HS-5. For RPMI 8226 cells in these conditions, pro-apoptotic protein expression in the cells was significantly greater in PIN/BTZ treatment versus the control (Figure 5c,d). Although the PIN/BTZ treatment effects were not as drastic in these MM cells co-cultured with HS-5 cells, the combination treatment resulted in increased proapoptotic expression in both co-cultured MM cells and MM cells cultured alone. Our data suggest that this combination treatment induces apoptosis in MM cells even with resistance from interactions with BMSCs. 

In clinical applications, BTZ is generally administered at a maximum dose of 1.3 mg/m^2^, though a dose of 1.6 mg/m^2^ has also been studied [18]. Depending on the severity of peripheral neuropathy symptoms, dosage is recommended to be decreased to 1.0 mg/m^2^ or 0.7 mg/m^2^ or discontinued altogether [18]. A clinical trial comparing BTZ/dexamethasone treatment to reduced dose BTZ/thalidomide and dexamethasone treatment showed the reduced dose BTZ treatment to have a greater overall complete response plus very good response in newly diagnosed MM patients, along with decreased peripheral neuropathy severity [56]. This illustrates the benefits of a synergistic drug application such as improving treatment effectiveness and requiring lower doses to minimize side effects in patients and reduce development of resistance. The combination of PIN/BTZ in this study shows potential as a synergistic anti-myeloma drug combination through its targeting of apoptosis and oxidative stress in MM cells. Further research of this treatment as well as other novel drug combinations is essential for the identification and application of optimized drug treatments that provide the most benefit to patients.

## 4. Materials and Methods

### 4.1. Chemicals and Reagents 

RES, PIN, PIC, BTZ, and BSO were purchased from Cayman Chemicals (Ann Arbor, MI, USA). Fetal bovine serum (FBS), penicillin-streptomycin solution, and PrestoBlue Cell Viability Reagent were purchased from Thermo Fisher Scientific (Grand Island, NY, USA). The FITC Annexin V Apoptosis Detection Kit was purchased from BD Biosciences (San Jose, CA, USA). NAC and the DCFDA/H2DCFDA Cellular ROS Assay Kit were purchased from Abcam (Cambridge, UK).

### 4.2. Cell Culture

Human MM cell lines (RPMI 8226, U266 and NCI-H929), human bone marrow stromal cell line HS-5, cell culturing medium RPMI-1640, and dimethyl sulfoxide (DMSO) were purchased from the American Type Culture Collection (ATCC; Manassas, VA, USA). The RPMI-1640 medium for RPMI 8226 and HS-5 cells was supplemented with 10% FBS and 1% penicillin-streptomycin. RPMI-1640 medium for U266 cells was supplemented with 15% FBS and 1% penicillin-streptomycin. RPMI-1640 medium for NCI-H929 cells was supplemented with 10% FBS, 1% penicillin-streptomycin, and 0.05 mM 2-mercaptoethanol. Cells were passaged every 2 to 3 days and maintained at their appropriate concentrations based on ATCC instructions. Human peripheral blood mononuclear cells were purchased from ATCC and cultured in Hank’s Balanced Salt Solution without Ca^2+^ or Mg^2+^ (ATCC) supplemented with 10% FBS. All cells were maintained in a humidified incubator at 37 °C and 5% CO_2_.

### 4.3. Cell Viability Assays

MM cell viability was assessed with PrestoBlue assays (Thermo Fisher Scientific). Stock solutions for all compounds were made using DMSO as the solvent. Compounds were further diluted in cell culture media immediately prior to cell treatment. MM cells were treated with increasing concentrations of RES, PIN, and PIC (0, 5, 10, 20, 30, 50 μM) as well as BTZ (0, 1, 2.5, 5, 10, 20, 25 nM). DMSO less than 0.05% was used as a vehicle control. In 48 well plates, MM cells were added at 40,000 live cells per well with a total 200 μL volume per well. Each treatment was done in triplicates. Plates were incubated at 37 °C and 5% CO_2_ for 24, 48, or 72 h. 10% of Presto Blue Cell Viability Reagent was added 1 h before fluorescence was measured with a Synergy HT plate reader (BioTek Instruments Inc., Winooski, Vermont, USA) at a 545 nm excitation and 590 nm emission. Fluorescence was normalized to the vehicle control. Half maximal inhibitory concentration (IC_50_) values were calculated for each compound using the “[inhibitor] vs. normalized response” setting on GraphPad Prism 9.3.1. Three independent experiments were conducted.

### 4.4. Synergism Identification

To determine possible synergistic combinations of treatments, various combinations of 2-drug treatments (BTZ with a RES derivative) were assessed for their effects on RPMI 8226 viability using PrestoBlue assays. RES derivatives and BTZ were screened for their cytotoxic effects in constant ratio combinations of 1:1000 BTZ to RES derivative (0 nM/0 μM, 5 nM/5 μM, 10 nM/10 μM, 20 nM/20 μM, 30 nM/30 μM, 50 nM/50 μM) as well as 5, 10 and 20 μM concentrations of RES derivatives with 1.25, 2.5, 5, 10 and 20 nM concentrations of BTZ. An amount of 5 and 20 μM concentrations of PIN with 1.25, 2.5, and 5 nM concentrations of BTZ was selected for confirmation of synergistic effects.

Data from the viability assays were analyzed using CompuSyn (Ver 1.0) software which calculates combination index (CI) values to determine whether a combination is synergistic, additive, or antagonistic [57]. A CI value of less than 1 would indicate a synergistic drug combination. The 5 nM BTZ and 5 μM PIN combination was selected for further analysis as a synergistic combination. The effects of the selected synergistic combination treatment and its individual components were assessed using 48 well plates in the MM cell lines RPMI 8226, U266, and NCI-H929. This combination’s effects on normal PBMC cells were also examined using the same cell viability assay. Treatments were done in triplicates, and three independent experiments were conducted.

### 4.5. Western Blotting

RPMI 8226 cells were treated with 0.05% DMSO (vehicle control), 5 μM PIN, 5 nM BTZ, and PIN/BTZ combination in 6-well plates at 1.2 × 10^6^ cells and 6 mL of media per well. After 24 h, cells were lysed, and protein was extracted using RIPA lysis buffer (Thermo Fischer Scientific Inc.). The Pierce BCA Protein Assay Kit (Thermo Fisher Scientific Inc.) was used to quantify protein samples, which were loaded at 20 μg per well into a 10 well 4–20% Mini-PROTEAN TGX precast gel (Bio-Rad Laboratories, Hercules, CA, USA). The gel was run at 110 V for one hour. Protein was transferred to Immun-Blot PVDF membranes (Bio-Rad Laboratories) for 7 min at 2.5 A using the mixed molecular weight setting on the Trans-Blot Turbo transfer system (Bio-Rad Laboratories). The membrane was washed with PBST (1X PBS with 1% Tween 20) three times for five minutes each. The membrane incubated in PBS-based Odyssey blocking buffer (LI-COR Biosciences, Lincoln, NE, USA) in 4 °C for an hour. Primary antibodies for cleaved caspase-3, cleaved PARP1, and α-tubulin (Abcam) were diluted 1:1000, 1:1000, and 1:5000, respectively, in Li-Cor blocking buffer. The Oxidative Stress Defense Western Blot Cocktail (Abcam), which includes catalase, smooth muscle actin, SOD1, and thioredoxin antibodies, was diluted 1:250. Membranes were incubated in this primary antibody solution overnight at 4 °C. The membrane was washed with PBST three times for five minutes each and incubated with secondary antibodies for 30 min. The membrane was washed with PBST again. The membrane was visualized on the Odyssey CLx Imaging System (LI-COR Biosciences, Lincoln, NE, USA), and intensity of protein bands was quantified using Image Studio Version 5.2 (LI-COR Biosciences).

### 4.6. Flow Cytometry Analysis

RPMI 8226 cells were treated for 24 h the same way as described in the Western blotting procedure. Following the 24 h treatment, the cells were isolated, washed in PBS, and then resuspended in 1 mL of binding buffer from the FITC Annexin V Apoptosis Detection Kit (BD Biosciences). In 3 replicates per treatment group, 100 μL of these cells were stained with 5 μL of both FITC Annexin V and 7-AAD dyes from the kit. Duplicates of controls were also made, which consisted of unstained cells, FITC-Annexin V only stained cells, and 7-AAD only stained cells. The tubes were incubated in the dark for 15 min at room temperature on a benchtop shaker. Prior to loading into the BD Accuri C6 Flow Cytometer (BD Biosciences), 400 μL of the binding buffer was added to each sample. The samples were run through the cytometer at medium speed to read a total of 10,000 units. Cell populations were gated and quantified using BD Accuri C6 software (Ver 1.0, BD Biosciences) to determine the percentages of normal and apoptotic RPMI 8226 cell populations.

### 4.7. Oxidative Stress Analysis

RPMI 8226 cells were pretreated with 0 mM or 1 mM NAC for 1 h prior to treatment with 5 μM PIN and 5 nM BTZ, with cell viability assessed following 24, 48 and 72 h as described in cell viability procedure. RPMI 8226 cells were pretreated with 0 mM or 1 mM BSO for 24 h prior to PIN and BTZ treatment, and cell viability was assessed after 24 h. PIN/BTZ treatment effects on production of ROS in RPMI 8226 cells were measured with the DCFDA/H2DCFDA cellular ROS assay kit (Abcam) following the manufacturer’s protocol. After 24 h treatment, fluorescence was read at 485 nm excitation and 535 nm emission using a Synergy HT plate reader. Western blotting was used to analyze expression of oxidative stress defense proteins catalase, SOD1, and thioredoxin in RPMI 8226 cells treated with PIN and BTZ for 24 h. Three independent trials per experiment were conducted.

### 4.8. Transwell Model

To address the effect of bone microenvironment on MM cell resistance to drug treatment, the apoptotic effects of the 5 μM PIN and 5 nM BTZ combination on RPMI 8226 cells were further studied using the Transwell model, where RPMI 8226 cells were co-cultured with HS-5 cells using 6-well Transwell plates with 0.4 μM polycarbonate membrane inserts (Costar, Corning Inc., Corning, NY, USA). In these 6-well Transwell plates, HS-5 cells were added at 2.5 × 10^5^ cells per well in 2.5 mL of media and allowed to adhere to the basal chamber for 24 h. Then, RPMI 8226 cells were added to the apical chamber of the Transwell insert at 5 × 10^5^ cells per well in 1.5 mL of media, to which the drugs were added. After 24 h, the RPMI 8226 cells were isolated, protein was collected and quantified, and Western blots were run to analyze proapoptotic protein expression as described in the Western blotting procedure.

### 4.9. Statistical Analysis

One-way analysis of variance and Dunnett’s multiple comparisons tests were run using GraphPad Prism 9.3.1 software (GraphPad Software, Inc., San Diego, CA, USA) to determine statistical significance. Results were presented as mean ± SD. A *p* value < 0.05 was considered statistically significant.

## 5. Conclusions

In this study, we observed RES, PIN, and PIC cytotoxicity in MM cells and identified the combination of 5 μM PIN and 5 nM BTZ as synergistic in reducing the viability of MM cells. This combination was shown to induce apoptosis in RPMI 8226 cells cultured alone as well as in RPMI 8226 cells co-cultured with bone marrow stromal HS-5 cells in the Transwell model, signifying the efficacy of the treatment even with increased drug resistance from HS-5 interactions. Our data suggested oxidative stress to be induced by the treatment as well. In studying the effects of RES derivatives on MM cell viability, as well as assessing synergism between RES-derivative PIN and the already implemented drug BTZ, we learned more about the drugs’ cytotoxic efficacies and possible MM-targeting mechanisms. Continuing with more extensive assessments of phytochemicals in combination with approved drugs like BTZ could lead to the identification of combinations with greater synergistic effects than those identified in this study. Researching such topics in vitro and subsequently in vivo can contribute to the development of future drug treatments that more effectively target MM cells, reduce relapse, and improve the wellbeing of people with MM.

## Figures and Tables

**Table 1:** IC_50_ values of RES derivatives and BTZ for effects on MM RPMI 8226 cell viability 24, 48, and 72 h following drug treatment.

	24 h	48 h	72 h
RES	51.49 ± 5.09 μM	26.95 ± 1.92 μM	13.19 ± 0.82 μM
PIN	51.38 ± 3.47 μM	25.78 ± 1.70 μM	13.21 ± 0.69 μM
PIC	32.33 ± 2.83 μM	18.57 ± 1.25 μM	13.82 ± 0.88 μM
BTZ	8.86 ± 0.82 nM	3.31 ± 0.33 nM	2.05 ± 0.22 nM

**Table 2:** Combination index (CI) values based on PIN and BTZ cytotoxic effects in MM RPMI 8226 cells. CI < 1 indicates synergism as determined by CompuSyn (Ver 1.0) software. The combination of 5 μM PIN and 5 nM BTZ is synergistic at all time points.

PIN (μM)	BTZ (nM)	24 h	48 h	72 h
5	1.25	23.1299	2.84814	1.82595
5	2.5	4.98265	2.19354	1.8127
5	5	0.97987	0.89462	0.90181
20	1.25	4.04991	1.85988	1.55874
20	2.5	5.21424	2.34567	1.91552
20	5	1.07322	1.10315	1.3671

## Data Availability

Not applicable.
